# On Apoplexy

**Published:** 1802-07

**Authors:** 


### To Dr. BRADLEY.

SIR,

AS the Controverfy refpecting Apoplexy feems to be drawing to a conclufion, a difpaffionate Obferver may be permitted to offer a few remarks as they occurred on perufing the papers on this fubject in the Medical and Phyfical Journal. I have not feen either Dr. Langflow's or Mr. Crowfoot's Publications on Apoplexy; I believe them both to be gentlemen of eminence in their profeffion, and highly refpected by their particular circles of friends. In offering the following loofe obfervations therefore, I fhall endeavour to avoid giving umbrage to either party, and attempt to fhew, that controverfy may be engaged in without infringing the *golden rule.* I beg leave however to obferve, as an apology for my concealment, that there appears to me to be no impropriety in wearing a mafk, fo long as my conduct is fufficiently refpectful to all parties. But it behoves an anonymous writer, to be peculiarly circumfpect and temperate in his expreffions; becaufe every wound which he inflicts on his adverfary, may be compared to that made by the cowardly affaffin, who lurks in the gloom of night, that he may ftab, with more fecurity, the man whom he dares not face in open manly combat.

The difputed points between Dr. L. and Mr. C. appear to be, whether emetics may be fafely adminiftered in apoplexy? and and whether compreſſion of the brain from an extravaſated fluid (I adhere to the etymology of the word) be always the cauſe of apoplexy. Theſe are points of too great importance for me to determine; but after having taken a ſlight review of ſome of the opinions offered upon this occaſion, I ſhall venture to give my own.

In No. xxxv. of the Medical Journal, Mr. Davies gives two caſes of apoplexy, both of which were probably beyond the reach of art. The firſt of theſe appeared, on diſſection, to have been a caſe of oppreſſed brain; whether the ſecond caſe was genuine apoplexy, does not ſeem clear; but perhaps a treatment ſimilar to that of the firſt, would have been leſs liable to objection. In No. xxxvi. Mr. Wilkinſon, with great propriety, defends the character of the benevolent and judicious Dr. Fothergill, a man to whom ſcience ſtands indebted, and whoſe memory is embalmed in every humane breaſt. The caſes of puerperal apoplexy, related by Mr. W. appear to have been the eclampſia gravidarum of Sauvages, an alarming and fatal diſeaſe. In an inſtance of this kind, I regret having had recourſe to embryulcia, when perhaps a few hours might have been waited with ſafety and advantage. Delivery appeared to have no effect in ſhortening the convulſions, which continued with dreadful violence for forty-eight hours. During the intervals of ſpaſms, ſhe laid in a ſtate of profound coma, from which ſhe at length awoke as from a deep ſleep, unconſcious of what had paſſed. She was a ſtrong, healthy young woman, and this was her firſt pregnancy. In two ſucceeding labours which I attended, no untoward circumſtance occurred, and ſhe recovered her ſtrength in all of them very rapidly. In the convulſions, daſhing with cold water was principally truſted to, as bleeding could not be employed, owing to her violent ſtruggles. Camphor and opium were alſo adminiſtered with apparent good effect; though it may not be improper to inſert here, a caution reſpecting the uſe of opium in this diſorder, as Dr. Hamilton of Edinburgh, aſſerts, that every patient died to whom opium had been exhibited. The termination of the caſe of apoplexy in dropſy, related by Mr. W. may, perhaps, admoniſh us to be more cautious in the uſe of the lancet, and rather to repeat the evacuation, if requiſite, than to take away the whole quantity at once.

Mr. Atkinſon's caſe, No. xxxvii. appears to me not to have been genuine apoplexy, but an inſtance of *determination* to the brain, or *engorgement*, ſuch as ſo frequently occurs in the advanced ſtate of typhus when it terminates fatally. What is the peculiar ſtate of the brain in ſuch inſtances, and in what particulars it differs from apoplexy is uncertain; but diſſections of of typhus cafes frequently fhew us inflammation, fuppuration, and even mortification, of the brain*.

In Mr. A's cafe, the prudent application of a blifter, was attended with more immediate fuccefs than we are in general gratified with in fuch defperate cafes. I fhould alfo fufpect, that the cafe of a young lady, related by Mr. A. was rather an inftance of hyfteria than of apoplexy. In Page 364, Mr. A. afks, "Are frothing at the mouth, fpafms of the mufcular fyftem, &c. &c. as reprefented by Mr. Wilkinfon, the diagnoftic fymptoms of apoplexy? Are not they decifively characteriftic of epilepfy?" In anfwer to this query it may be obferved, that convulfions *sometimes* occur in apoplexy, and that *spuma oris*, is noticed by a variety of authors as occurring in this difeafe, Callifen Princip. Chirurg. Sprengel Handbuch der Pathologie, &c. Bellini obferves, "Spuma etiam circa os aliquando contingit, fed iftud rarius multoque rarius fpuma per Nares redditur. De Morb. Capitis." This is, however, pofitively denied by Cælius Aurelianus, who fays, "Epileptici quidem totius corporis *conductione* agitentur, & fpumas agant, *apoplecti* vero nunquam." Apoplectics appear to be more or lefs in a profound fleep, and the *joints remain flexible.* Mr. A. gives an inftance of apoplexy fucceeding palfy; but many inftances occur wherein a confiderable degree of torpor, and even of palfy, precede apoplexy. A cafe was related by Dr. Cullen, in his Lectures, of a perfon who was accuftomed to hang his head over fermenting liquors, and who became paralytic, and died apoplectic fome time afterwards.

In No. XXXVIII. Pyrrho enters the Palæftra, and wields the pondrous cæftus with dexterity and eafe; nor fhall I prefume to oppofe my puny efforts to fo powerful a combatant, left the fate of Dares fhould await me. Notwithftanding, however, that Pyrrho fets out very properly, with lamenting the virulence and perfonal invective which have been fo much fhewn in the prefent controverfy, he falls into the fame error, and treats Dr. L. in a more difrefpectful manner than is confiftent with that fpirit of candour which he profeffes to poffefs. Does not Pyrrho likewife fpeak in a "*vaunting manner*" on feveral occafions? And in particular, in his office of gentleman ufher, does he not too cavalierly introduce gentlemen to Dr. L's acquaintance, with whom the Doctor is probably intimately acquainted?— But, let me beware of cenfure.

Pyrrho's obfervations upon the effects of Emetics, are judicious; and I agree with him in thinking, that, owing to the diminifhed

* S. G. Vogel's Handbuch der Pract. Arzneywiffenfch.

diminiſhed energy of the heart, leſs blood is ſent to the brain during the niſus to vomit: but I differ from him in ſuppoſing, "that in the act of vomiting, the ſtate of the brain is rather that of depletion than plenitude." If we attend to what happens during the inſtant of vomiting, we find the whole frame violently agitated; the primary muſcles of reſpiration are, as it were, ſpaſmodically affected to elevate the thorax; the muſcles alſo inſerted into the thorax and ſcapulæ, though not immediately ſubſervient to reſpiration, are brought into action to ſupport and fix thoſe parts. Even the muſcles of the neck, (not to mention thoſe of the larynx and pharynx) the maſtoid and ſcalene, which laſt ariſe from the tranſverſe proceſſes of the cervical vertebræ, and are inſerted into the firſt rib, are excited to act with violence to ſupport the ribs, which are attempted to be forced down by the abdominal muſcles that now act as antagoniſts. Theſe violent efforts, by preſſing the abdominal viſcera, expedite the return of blood by the inferior cava, which from its quantity, appears to give a momentary oppreſſion to the right ſide of the heart: this, together with the action of the muſcles, retards the blood returning by the jugulars, which appear ready to burſt with their contents; the face aſſumes a purple hue; the eyes project, and the countenance appears turgid. From hence, it is probable, that in vomiting, the blood is merely delayed in its return from the brain; and if we conſider the nature of the ſinuſſes, it ſeems reaſonable to conclude, that the increaſed turgeſcency is rather in the branches and trunks of the jugulars than within the cranium. We know that the ſkull is ſo completely filled with its contents, that no cavity remains; even the ſides of the ventricles are cloſely preſſed together. After death we find no void ſpace: indeed, the brain is ſo cloſely *packed*, that when, to remove the upper portion of the cranium, the bones are nearly ſawn through, and a degree of force is applied with a levator, they fly aſunder with a ſpring; and it is abſolutely impoſſible, with all our art, to *cram* the brain into the ſame ſpace it occupied before. It is this firm preſſure of the parts upon each other that preſerves the veſſels from rupture; for they are thinner in their coats here than in other parts of the body. If the brain could be removed by a charm, and the veſſels be left ſuſpended, though without ſuffering any injury, it is probable that rupture would be the immediate conſequence. If a portion of the ſkull be removed by the trephine, a pulſating motion, an elevation of the brain is obſerved, merely becauſe the reſiſtance of the bony arch is removed: it does not occur in the natural ſtate, and would probably be productive of injury.

In irritable and hyſteric females, more powerful effects are often produced by a flight inequality, fuddenly arifing in the circulation, than from the ftraining of an emetic ; thus a trifling alarm, fuch as fcarcely accelerates the pulfe 6 or 8 beats in a minute, will produce a violent pulfating pain in the head for many hours ; yet we frequently find fuch head-achs, efpecially when connected with a difordered ftomach, alleviated or removed by an emetic, which acts, not fo much by unloading the ftomach as by producing a more equable circulation. In an afthmatic paroxyfm, where the appearance of determination to the head, or at leaft of obftruction to the return of the blood, is often very alarming, we do not find an emetic productive of dangerous effects. Even in hæmoptyfis*, emetics have been employed for the cure, if not with complete fuccefs, at leaft with impunity.

I cannot prefume with Dr. Moffman, to think that no practice is fraught with greater danger than the adminiftration of emetics during the attack of an apoplexy ; becaufe they have been tolerated, and even recommended "*by the higheft profes-sional authorities.*" In addition to the teftimonies adduced by Pyrrho, I fhall only add, that Baglivi, who divides apoplexy into fanguineous and pituitous, obferves, "Arcanum in fanguineis eft phlebotomia. In pituitofis contra emeticum, aut purgans vehemens. Sunt qui apoplexia (pituitofa fcilicet) liberati funt, haufto fingulis menfibus vomitivo ex infufo prædicto (infus. croc. metal. cum vino.") Aretæus does not recommend emetics, but obferves, "if the *facred purge* fhould excite vomiting, it is not to be reftrained, becaufe it evacuates pituita, the caufe of the difeafe, and roufes the patient by imparting a degree of vigour." Our venerable countryman, Dr. Heberden, in his valu.ble legacy (Commentarii de Morbor. Hiftoria) appears to be much of the fame opinion. "When the patient," he fays, "is affected with naufea, an infufion of carduus benedictus may be given, but no ftronger medicine."

* The two alarming cafes of epiftaxis, given by Pyrrho, which were put a ftop to by the motion of a carriage, are ufeful in a practical view. The fame good effects have been fo frequently obferved in hæmoptyfis, that geftation has often been employed with fuccefs for its cure. It produces a more equable circulation by determining to the furface, and thus removes internal congeftion. The fame good effects, and even more powerful ones, if we may judge from the coftivenefs induced, are obtained by failing. See Gilchrift on Sea Voyages. Dr. Cullen, in his Lectures, mentioned the cafe of a perfon in hæmoptyfis, who fet out from Edinburgh to go to Briftol, and before fhe had been a day on the road, the difcharge ceafed ; when fhe ftopped, the blood returned, and difappeared again when fhe fet out ; arriving at Briftol, it returned again.

It is, however, to be doubted, whether a dofe of ipecac. or what is preferable, of vitriol. zinc. will not unload the ftomach with lefs exertion than can be done by the feeble efforts of a naufeating medicine. Boerhaave, among the general evacuants to be ufed in this difeafe, mentions vomits and ftrong purges; though he adds, there is fomething uncertain in their action. Vanfwieten, alfo, in his Comment. upon this Aphor. (1026,) obferves, that emetics ought not to be condemned in this difeafe, and are often ufeful, becaufe they evacuate pituita; though he afterwards thinks purgatives lefs objectionable. Dr. Cullen, in his Firft Lines (fect. 1134,) objects to vomiting; but in my manufcript notes, taken from his Lectures, in 1788, he thus expreffes himfelf: "This (vomiting) hath fometimes been practifed with fafety. It is difficult to explain how full vomiting is employed without increafing the congeftion; yet here, facts are in part againft our theoretic fears." Lieutaud remarks, "Dans l'apoplexie fereufe, on doit *commencer* parles *vomitifs*, *à grande dofe*; & s'ils ne produifent aucun effet, on peut en venir à la poudre d'Algaroth." (Precis de la Medicine.) Profeffor Callifen recommends the exhibition of an emetic, together with general and topical bleeding, to avert the threatening fymptoms of apoplexy. From the authorities above quoted, we may prefume that emetics are not fuch dangerous remedies in this difeafe as has been reprefented; and if I may prefume to offer an opinion upon the fubject, I fhall add, that an emetic would have been a judicious remedy in the cafe in difpute; for, all the time this remedy was prefcribed by Mr. C. the peculiar affection of the brain was removed: in fact, there was no apoplexy remaining—the patient had recovered her fenfes; for *fhe complained* of head-ach and ficknefs.

In page 432, Pyrrho proceeds to the confideration of apoplexy, as at all times depending upon effufion of blood or ferum in the brain; an affertion which P. obferves, is haftily hazarded by Dr. L. and which is at variance with found analogy, and the evidence of the beft Medical Records. I muft here differ entirely from Pyrrho, in thinking this an hafty opinion, as it has been univerfally received for ages*. The divifion of apoplexy into fanguineous, ferous, and pituitous, which is almoft as old as the annals of medicine, feems to prove, that

* The opinion that apoplexy arifes from extravafation, though untenable in its full extent, is, even at the prefent day, more univerfally received than any other. Dr. Flemyng in his Phyfiology, afferts, that "of an hundred apoplexies, ninety-nine will be produced by extravafation of blood within the cranium, or an accumulation of lymph or ferum within the ventricles of the brain."

Phyficians have, very generally, attributed apoplexy to an extravafation of one or other of thefe fluids. If evidence were neceffary to fupport this opinion, Dr. L. might bring an hoft. It has, however, been much weakened by the obfervations of later anatomifts, who have fhewn, that, in many inftances, no extravafation was to be feen; indeed, diffection has thrown no light upon that peculiar ftate of the brain which exifts in apoplexy; nor has it fhewn any organic difference between this difeafe and epilepfy, or between it and another, which more clofely refembles apoplexy, *concuffion of the brain.* In many inftances of apoplexy, diffection has difcovered no vifible injury of the brain; and on the contrary, large ulcers, abfceffes, confiderable collections of water, tumours, bony excrefcences, and offifications of the membranes have been obferved, though no apoplectic fymptoms had occurred during life. Nay, the furface of the brain has been feen almoft inundated with blood, yet apoplexy had not enfued. Thefe facts, however, only prove, that extravafation is not fo generally the caufe of apoplexy as has been imagined; and that the brain can fupport, without much injury to its functions, a very confiderable degree of preffure, when applied in a flow and gradual manner. It is not neceffary to prove, that preffure on the brain can produce apoplexy, becaufe we fee frequent inftances of it in depreffions of the fkull from external injury; and we often fee the apoplectic fymptoms *inftantly* removed by the application of the trephine. In a cafe likewife where a portion of the fkull had been removed, as related by Boerhaave, a certain degree of preffure upon the brain with the hand, always induced apoplexy. When effufion is difcovered in the brain of perfons carried off by apoplexy, we may be allowed to fuppofe that they ftand in fome relation to each other of caufe and effect: it cannot arife in every inftance from exudation after death.

The cuftom of treating difeafes according to their names, without regard to their nature, or to the particular conftitution of the patient, has been productive of incalculable mifchief; and in no difeafe has fuch a practice produced more baneful effects than in apoplexy. Some years ago the *cant word* among practitioners was, *Empty the vessels*; at prefent the cry is, *Support the system.* Both extremes have been run into in this difeafe; for whilft one party ordered blood to be taken away, not in *streams*, but in *rivers*, the oppofite party forbad a fingle drop to be loft. The truth will probably be found, as in many other inftances, *somewhere* between thefe extremes. Boerhaave obferves, that no general rule of cure can be laid down in apoplexy; and this feems more applicable to the ufe of blood-letting than of any other remedy. Venæfection was confidered confidered by the antients as a doubtful remedy, and they give many cautions refpecting its ufe, none of which are more judicious than thofe of Aretæus. Thus he obferves, " It is not eafy to determine the quantity of blood to be drawn; if too much be taken away, the patient is killed ; if too little, no benefit is derived from this *great remedy*." But he adds, "it is better to err by taking away too little than too much, becaufe the operation may be repeated. After blood-letting, acrid glyfters are to be ufed, and then the *facred purge*." Celfus fays, " Sanguinis detractio vel occidit vel liberat." Ætius alfo, fpeaking of venæfection, fays, " Detractio autem fiat e dextra manu, & copia ejus qui detrahitur, dividatur. *Commovere* enim oportet tantum, non exolvere vires & calorem extinguere."

The following quotation from an ingenious Thefis on Apoplexy, publifhed at Edinb. in 1777, breathes the ardour of a young man, who would now, probably, when fobered by fo many years experience, fhrink to put it in practice. Speaking of v. f. in the fanguine apoplexy, he fays, " Neque ut nonnulli medici volunt parca, e. g. ad ℥viij. fed dupli, ut minimum, imo tripli, aut etiam quadrupli, detractio effe debet." And in a note, he adds, " Cullenus aliquot libras cum fructu detractas vidit." Pyrrho expreffes his doubts refpecting the utility of blood-letting in apoplexy in a very judicious manner ; and remarks, that of a number of cafes which he had feen treated by v. f. he has ftrong doubts " whether thofe that loft the greateft quantity of blood have not proved the moft fatal." When a perfon drops down in a fit, it is immediately referred to apoplexy ; the by-ftanders are alarmed ; all is trepidation and buftle ; and the firft thing which occurs to them is blood-letting, which, to pleafe them, is to be performed immediately. But inftead of haftily yielding, we ought to deliberate a little, and not take away blood merely becaufe the difeafe is apoplexy. We fhould confider what have been the exciting caufes, and what is the habit of body or powers upon which thefe have acted. If we know the peculiar habits of the patient, we have a clue to guide us ; if he be a ftranger, our indications muft be drawn from external appearances. In full, robuft habits, where violent exertions alternate with profound floth and a luxurious nourifhing diet; where the fanguine temperament is ftrongly marked by a full and active ftate of the veffels, bleeding may be immediately had recourfe to. But even here, it may be doubted whether the quantity taken away at once fhould exceed ℥viij, or ℥x ; it will be more prudent to adhere to the advice of Archigenes, (Ætii tetralib. de fyderatione) and attend during the evacuation to the ftate of the pulfe, the colour of the face, and to the refpiration; and if thefe indicate no harm to have enfued, the abftraction may be repeated with the fame caution. We ought not to be governed by the hiftories of fuch aftonifhing quantities of blood, apparently inconfiftent with the continuation of life, which have been loft with fafety, and even with advantage. Thus Burferius *quotes from Lancisi, in support of the use of copious bleeding, even in old persons,* the cafe of a very old merchant threatened with apoplexy, who was fnatched from impending danger, and relieved by a fpontaneous hæmorrhage from the nofe to the amount of eleven pounds, and who was entirely freed from the complaint fifteen days afterwards by the eruption of four pounds more.

In the early part of my practice I recollect being called to a woman, who, in little more than two years, had three attacks of genuine apoplexy, from which fhe perfectly recovered. A fourth attack fucceeded by hemiplegia, in which the *left side* was affected, and the levator palpebræ of the *right eye*. Bleeding to the amount of ℥x. was had recourfe to each time, with glyfters compofed of a folution of purging falts, and ol. terebinth. which appeared of fervice. She was a low, but remarkably ftout, corpulent woman, about fifty, with a fhort neck and very florid complexion, accuftomed to indulge her appetite in grofs eating, particularly hot fuppers of animal food. In perfons of a relaxed debile habit, though attended with corpulency and the appearance of plethora, general blood-letting will require caution. This is particularly requifite where there appears to be exhauftion of the nervous or fenforial power, produced by violent or continued excitement of the brain, whether arifing from deep thought and anxiety, or violent emotions, as furprize, &c. Many fuch inftances occur in Hiftory, but none is more affecting than that of the fond mother, who died in the embraces of her fon, who had unexpectedly efcaped from the flaughter of Cannæ. In fuch cafes, depreffing powers are evidently hurtful.* Frictions, moderate warmth, the judicious application of ftimuli, as of volatiles to the nofe, blifters to the head or temples, or tincture of cantharides to the legs or feet, appear to be the moft proper means; if the power of fwallowing remains, a little fpirit and water, or a few drops of t. opii to be occafionally repeated, may be adminiftered; where, with general debility, there appears to be congeftion in the head, cupping glaffes may be applied to the occiput, with or without fcarification. Thefe were ftrongly recommended by the antients, but appear now to be fallen into difufe; though they are much preferable

* Among ftimuli to be ufed, Bellini mentions pilorum evulfiones fubitas.

ferable to leeches, as being more manageable and making a more fudden depletion. In fhort, in making evacuations, we ought to confider, that if caufes, which diminifh the energy of the brain and induce a fudden ftate of collapfe, be capable of producing death when in a high degree, or apoplexy in a lower, in fuch inftances every debilitating power muft be carefully fhunned. This is more clearly expreffed in the following quotation from a late publication, replete with valuable practical information. "Another fpecies of apoplexy, which affects perfons of a weakly conftitution, who are pale, thin, and emaciated, who have been depreffed by forrow or misfortune, though it feldom occafions inftantaneous death, may yet render the remainder of life burthenfome, by difabling the limbs, and enfeebling the memory. Under fuch circumftances, whether we fuppofe congeftion of blood and effufion of lymph to take place from a languid, irregular circulation, or the communication between the blood veffels and nerves to ceafe partially from a ftill higher degree of debility, it will be manifeft that the remedies above-mentioned, (bleeding, cupping, blifters, and evacuants) at leaft that large or repeated bleeding, with the ufe of emetics, purgatives, &c. muft be injurious, if not fometimes fatal *."

In page 334, Pyrrho obferves, "In no inftance, either in health or difeafe, is the volume of blood equal to the collective areas of the veffels; for upon killing any animal, in the fulleft and beft health, it will be found that the arteries are wholly empty, and that the blood in the fyftem will barely fill the veins." To this it may be replied, that Pyrrho confiders the veffels too much in the light of elaftic canals, and does not advert to their living, mufcular power. In the living body the veffels are always full; and as a proof of their mufcularity, may be adduced the power they poffefs of accommodating themfelves to the quantity of their contents. When blood is fuddenly loft in large quantity, as by a wound, an uterine hæmorrhage, &c. the *tension* of the veffels is fo inftantly removed, that is, they cannot adapt themfelves fo immediately to their contents as is neceffary for health, that fainting or death enfues. After death, the irritability of mufcular parts continues for a confiderable time; and as the mufcularity of the arteries is more evident than that of the veins, of confequence they will retain their activity longer, and propel their contents

* Reports on the difeafes in London, by Robert Willan, M. D.

That peculiar ftate of the nervous energy, which has been called exhauftion, or "diminution of vitality," is not unaptly compared by Ramazzini to an eclipfe of the brain, which organ he terms Lunare fydus microcofmi.

tents into the veins, which yield to the diftenfion. It is from this caufe that the lymphatics are always found empty. In many cafes of fudden death, where the irritability is fpeedily, or inftantly deftroyed, the larger arteries are frequently filled with coagulated blood, called polypi. A curious inftance of this is related by Ramazzini, from P. Salius de Aff. Part. c. 4. in a girl who died fuddenly. On diffection, a polypous concretion was drawn from the aorta, "Non aliter quam gladius e vagina extrahi folet."

I hope Pyrrho will not take offence if I think him unreafonably captious, when at page 335, he requires Dr. L. to prove the exiftence of lymphatics in the brain. We cannot prove the vafcularity of the medullary part of the brain by injection; yet no perfon doubts that it poffeffes red veffels; and in thofe who have died apoplectic, on cutting into the medullary part, numberlefs red fpots, the mouths of veffels, are frequently obferved. We cannot, indeed, demonftrate the lymphatics, but their exiftence is fufficiently proved by analogy. The long chain of lymphatic glands, called Glandulæ concatenatæ, which accompany the blood-veffels to the bafis of the fkull, muft be fubfervient to the brain, or why do they terminate fo abruptly? What becomes of the vapour or fluid, fecreted within the ventricles? Were it not removed, hydrocephalus would enfue. The reafon that the lymphatics of the brain have not hitherto been difcovered, probably depends upon the minutenefs and the pellucidity of their coats; for I have no doubt, when future anatomifts detect them, that they will be found as numerous as the blood-veffels of the brain. The abforbing power alfo of thefe veffels will probably be found in inverfe proportion to their magnitude, and their activity will perhaps be further increafed by being placed in the centre of nervous energy. I am therefore of opinion with Dr. L. that the lymphatics of the brain may from thefe caufes, be quicker in their action than in other parts of the body; and I do not hefitate to declare my conviction, that extravafated fluids would be abforbed in any quantity from the brain with as much certainty as is a flight ecchymofis in another part, were not the veffels *paralyzed*, as it were, by the preffure. Dr. L's hypothefis refpecting the progreffive abforption of fluids effufed in the brain is certainly ingenious; but though Dr. L. cannot demonftrate his opinion, yet it will be, perhaps, equally difficult for Pyrrho to contradict it. In p. 336, Pyrrho fuggefts that apoplexy may frequently be feated in the lungs, rather than in the head. We know that tumours in various parts, by preffing upon the large veffels, and occafioning a greater quantity of blood to flow to the head, have occafioned apoplexy. Dr. Cullen related the cafe of a lady who had a fteatoma in the abdomen, which, when fhe

ſhe ſtooped, preſſed on the deſcending aorta, and immediately induced apoplexy. Similar inſtances may occur in the lungs; and ephialtes or incubus, which has been by ſome conſidered as a *slight apoplexy*, may ſerve as an inſtance, though its ſeat may, perhaps, be placed in the ſtomach rather than in the lungs. I cannot agree with Pyrrho in ſuppoſing that apoplexy may ariſe from a defective oxygenation of the blood in the lungs; for were it ſo, ought not apoplexy to be a frequent ſequela of aſthma? Dr. L. I think, very juſtly aſks, " Do not the countenances of thoſe perſons ſuppoſed to be prediſpoſed to apoplexy, indicate *excessive* rather than *defective* oxygenation ?" This ſeems to be the caſe, at leaſt in one ſpecies of this diſeaſe, the carus ab inſolatione or coup de ſoleil; it occurs moſtly in young robuſt habits who have uſed much exerciſe and been expoſed to a ſcorching ſun; and blood taken from the arm appears remarkably florid and hot*. We have an inſtance of apoplexy ariſing from the ſtomach in conſequence of poiſons, and a ſtill more ſtriking one is given by the ingenious and learned Dr. Ruſh, of Philadelphia, occaſioned by drinking cold water in hot weather, when the body is much heated; opium is the only certain remedy for it, ' from a tea ſpoonful to near a *table spoonful* (of tinct. opii) have been given in ſome inſtances, before relief has been obtained.' (Med. Inquiries and Obſervations, Vol. I.) An inſtance of this is alſo mentioned by the celebrated Weikard, one of the moſt zealous defenders of the *Brunonian* ſyſtem in Germany. A man who had fatigued himſelf much by an haſty journey, drank a large quantity of cold water, from which he felt unwell, and ſuddenly died of apoplexy; here, the debilitating powers of the cold water were firſt exerted on the ſtomach, and propagated from thence to the brain and nerves. " In this manner it is, he continues, that apoplexies are occaſioned by flatulent, fermenting, and indigeſtible foods. Thoſe alſo who are troubled with incubus from flatulence are much expoſed to apoplexy." (Med. Prackt. Handbuch auf Browniſche Grundsaze.)

Among the numerous diviſions of apoplexy, is included by many, the A. epidemica, as connected with certain ſtates of the weather ( Hippo. Aphor. 16, 23, Sect. 3.) It is noticed by Baglivi, Kirkland, and others. At Konigſberg, in Germany, in

* In Schmucker's (the celebrated Pruſſian Surgeon) Vermiſchte Chirurg. Schrift. it is ſaid that the king of Pruſſia, in a retreat by forced marches, loſt 300 men by this diſeaſe. It was obſerved that all thoſe who were let blood, died; placing the patient in the ſhade, expoſed to a current of air, waſhing him at the ſame time with cold water, or vinegar and water, was found moſt effectual. Wet cloths ought alſo to be applied to the head.

in the year 1784, of 1918 deaths, 71 were occafioned by apoplexy; and in the following year, the deaths amounted to 2201, of which number 76 were apoplectics. (Finke Verfucheiner allgem. Med. pr. Geographie) The idea of apoplexy being connected with feafon, has arifen from the cuftom of referring every fudden death to this difeafe. Pliny gives a long lift of fudden deaths, which he calls 'Summa vitæ felicitas,' though it is to be regretted that he has not given us a more particular account. Several fuch inftances are related by Morgagni, though it may be obferved, if thefe deaths were connected with a peculiar ftate of the weather, we fhould have expected fome ftriking fimilarity in the diffection of perfons who died in the fame month, on the fame day, and in the fame place, and of the fame difeafe; which, however, was not the cafe.

In forming a Prognofis in this difeafe, authors difagree very much refpecting fever when it fupervenes. Hippocrates remarks, that if a perfon be feized with apoplexy, he dies in feven days unlefs fever occurs; and this opinion has been followed by the generality of fucceeding writers. Thus, Profeffor Telle, of Berlin, in his Medicina Cl nica, after obferving that there is great danger when the power of fwallowing is loft, adds, 'it is alfo dangerous if fever does not foon appear.' Burferius alfo obferves, 'In fideratis, fi febris accedat, folutio fit,' though he modifies this confiderably afterwards. Dr. Heberden, on the contrary, denies that fever is ever productive of advantage in this or any other difeafe; and Dr. Whytt eftimates the danger by the quicknefs of the pulfe. From a cafe which lately occurred to me, I am difpofed to embrace the latter opinion. An old lady, aged eighty-five, tall, and remarkably thin, was affected with fudden faintnefs, from which fhe foon recovered, and ate with appetite a boiled egg; fhortly afterwards, it was difcovered that fhe had loft the power of one fide, the heat and feeling ftill remaining; her pulfe was about 76, and of natural ftrength. A degree of coma gradually came on, and her faculties began to fail. On the 4th day, the pulfe became remarkably full, ftrong, and vibrating, beating 100 in a minute, and continued gradually to increafe, for three fucceeding days, to 120 beats, when fhe died. This correfponds with an obfervation of Ballonius, who fays, that in all comatofe affections, efpecially lethargy and apoplexy, when a fmall pulfe becomes full and gradually increafes, the patient is foon carried off. Dr. Home feems to allude to this when he afks, 'Cur fenfu motuque decrefcentibus, pulfus fæpe validior fiat? Principia Medicinæ.

The refpiration alone affords a fallacious prognofis; for we have have inftances of deep fnoring, with frothing at the mouth, terminating favourably, while an eafy and almoft natural refpiration has ended in death.

I have thus prefumed to offer an opinion upon the important fubject of apoplexy, and flatter myfelf, that the gentlemen from whom I have ventured to differ, will pardon the liberty I have ufurped. I fhould alfo apologize to you, Sir, for having fo far tranfgreffed the limits of your excellent Journal; but as you poffefs the power, I hope you will freely ufe it, and expunge whatever appears irrelevant to the fubject.

I remain, &c.

